# Morphology of the Sella Turcica in Individuals With Different Skeletal Malocclusions in Upper Egypt Assessed Using Lateral Cephalometric Radiographs

**DOI:** 10.7759/cureus.63642

**Published:** 2024-07-02

**Authors:** Mostafa A Mohammed, Dina G Anis

**Affiliations:** 1 Orthodontics, Minia University, Minia, EGY; 2 Oral and Maxillofacial Radiology, Minia University, Minia, EGY

**Keywords:** egyptian individuals, lateral cephalograms, linear measurements, different skeletal malocclusions, st, sella turcica

## Abstract

Objective: To evaluate the morphology of sella turcica (ST) in individuals with different skeletal malocclusions in upper Egypt.

Materials and methods: 300 lateral cephalometric radiographs of adult patients of both sexes, varying ages from 18 to 30 years, were selected and divided into three equal groups, group (1): skeletal class I (control group), group (2): skeletal class II, and group (3): skeletal class III. Pre-treatment lateral cephalograms were taken from the archives of the Department of Orthodontics, Faculty of Dentistry, Minia University. The tuberculum and dorsum sella, the floor of ST, and posterior and anterior clinoid processes (ACPs) were drawn. The direct measurements such as diameter (APD), depth (D), and length(L) of sella were measured using Silverman and Kisling methods.

Results: A significant difference was found in depth (D) between class I and class II, with class II having a greater depth. Also, the largest diameter (APD) was found in the class III group. A significant difference was found in diameter (APD) between the two age groups (from 18 to 24 years and from 25 to 30 years)

Conclusion: The larger diameter values were seen in the skeletal class III subjects, while the larger depth values were observed in the class II subjects. The older age group (25-30 years) has a greater diameter than the younger one. Highly significant differences were found in length and depth between the sexes.

## Introduction

Several cranial landmarks have been identified to use as reference points for tracing cephalometric radiographs, which is one of the most important diagnostic tools for orthodontic treatment. These markers help identify how certain features, such as the mandible or maxilla, relate to the skull or each other. Examining these structures can help the orthodontist make the diagnosis, study an individual's growth using longitudinal structure superimposition, and assess the outcomes of orthodontic treatment [1].

When doing a neurocranial and craniofacial radiography examination, the sella turcica (ST) is a crucial component. Since the sella point is one of the most regularly used cranial landmarks for cephalometric tracing, the ST is a crucial anatomical component in orthodontics. The pituitary gland at the cranial base is located in the middle of the ST in this position. The ST is located on the intracranial surface of the sphenoid bone's body. The tuberculum sellae represents the anterior border of the ST, and the dorsum sellae represents the posterior margin. The ST surrounds the pituitary gland, and two anterior and two posterior clinoid processes (ACPs) protrude across the pituitary fossa. The ACP is a pyramid-shaped bony projection of the lesser wing of the sphenoid bone and forms part of the lateral wall of the optic canal. Between each ACP lies the ST, which holds the pituitary gland [2].

The size and shape of ST can be affected by a number of pathological diseases, including William syndrome, primary hypopituitarism, growth hormone deficiency, congenital lumbosacral abnormalities, and Seckel pear syndrome [3-6]. Additionally, severe craniofacial deviation, tooth agenesis, canine impaction, and people with cleft palate can all be strongly associated with this kind [7-9]. It is necessary to show that the ST has a normal morphology in order to identify the anatomical problem of the sella area. The typical pattern, nevertheless, differs from person to person. As a result, each race must be evaluated separately. That has been reflected in the written works [1,10,11]. The current study's objective was to evaluate and compare the size of ST in upper Egyptian subjects with various skeletal malocclusions and provide a new method to detect skeletal malocclusion through the size of ST.

## Materials and methods

Subjects

In a retrospective study approach, the pre-treatment lateral cephalograms of 300 adult patients were taken from the archives of the Department of Orthodontics, Faculty of Dentistry, Minia University, Upper Egypt, Egypt. The inclusion criteria were the patient’s age range from 18 to 30 years with no previous orthodontic, orthopedic, or surgical treatment, no craniofacial trauma, and no congenital anomalies. All lateral cephalograms that were not of high quality and not clear for interpretation were excluded.

Ethical regulation

This study was approved by the Research Ethics Committee of the Faculty of Dentistry, Minia University, Upper Egypt, Egypt (ID number: 794/2023). All radiographs were coded. 

Sample size

Before the study, the number of lateral cephalograms required in each group was determined after a power calculation according to data obtained from previous studies. 100 lateral cephalograms in each group were determined to provide 80% power for the one-way ANOVA test at the level of 0.05 significance using G Power 3.1 9.2 software.

**Figure 1:**
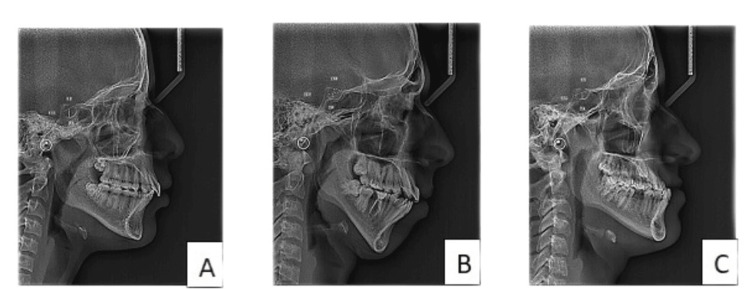
Linear measurements of ST in different groups: A) class I, B) class II, and C) class III ST, sella turcica

All lateral cephalograms were obtained using Planmeca Promax 3D Mid (Asentajankatu, Helsinki, Finland) with 66 KVp and 10 mA for 6.8 sec. The subspinal-nasion-supramental angle (ANB angle) was used to classify skeletal types into classes I, II, and III, with 100 pre-treatment lateral cephalograms for each group. The ANB angle illustrates how much of a difference there is between the skeletal jaws, regardless of which jaw is problematic. Class I skeletal bases were those with an angle of less than two degrees, Class II skeletal bases with an angle of more than four degrees, and Class III skeletal bases with an angle of less than zero degrees. In order to overcome the limitations of the ANB angle; A false value can be measured with an altered antero-posterior and vertical position of nasion, change in vertical face height, and alteration of SN plane. We used a linear measurement (Witt’s analysis) to determine the actual sagittal skeletal discrepancy (Figure 1) [1]. The Wits appraisal provides a credible reference to the severity of the antero-posterior skeletal disharmony of the jaws. They were divided into three equal groups: group 1: included 100 lateral cephalograms of patients with skeletal class I; group 2: included 100 lateral cephalograms of patients with skeletal class II; and group 3: included 100 lateral cephalograms of patients with skeletal class III.

Cephalometric analysis and the size of sella turcica

**Figure 2:**
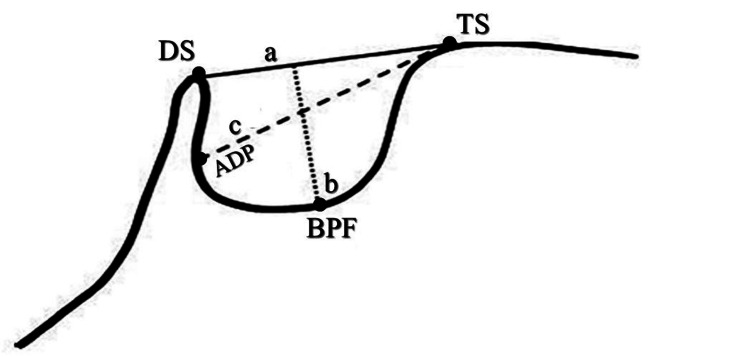
Normal ST morphology: TS, DS, and BPF a: length of sella (black line); b: depth of sella (dotted line); c: diameter of sella (dashed line) ST, sella turcica; TS, tuberculum sella; DS, dorsum sella; BPF, base of the pituitary fossa

Each cephalometric radiograph was traced with the web-based program for cephalometric analysis (WEBCEPH analysis) to determine the ANB angle and Witt’s analysis. After tracing with software, all tracing points were re-evaluated manually to ensure the accurate position of each point. The ST dimensions were measured by using the digital software Romexis 4.4.2.r. (Planmeca Oy, Helsinki, Finland). The tuberculum, dorsum sella, the floor of sella, posterior, and ACPs were determined. The methods of Silverman (1957) and Kisling (1966) were used to measure the linear dimensions of ST [12,13]. The mid-sagittal plane served as the location for all reference lines employed in the current study. The distance between the tuberculum sella and the tip of the dorsum sellae was used to determine the length of the ST. The ST's depth was determined by drawing a straight line from the top of the floor to its deepest point. Additionally, a line was drawn along the posterior inner wall of the fossa from the tuberculum sella to its farthest point. This was taken into consideration as the ST's antero-posterior diameter (Figure 2) [14].

Reliability of measurements

In order to reduce errors resulting from variations in the interpretation of the pictures by one operator, a set of 60 lateral cephalograms (20 radiographs from each group) were randomly chosen and retraced under identical circumstances three weeks later. The variance component estimates were obtained using the ANOVA model for one-way random effects. Commonly used as a gauge of measurement reliability based on these characteristics is the ICC. In this work, we evaluated the measurement consistency using the ICC [15]. The retraced radiographs have good consistency, as evidenced by the reliability measurements, which ranged from 0.99 to 1.00 (Table 1).

**Table 1:** Reliability of measurements of linear dimensions in the groups *Significant level at P-value <0.05

	All classes			Class I			Class II			Class III		
	Cronbach's alpha	ICC	P-value	Cronbach's alpha	ICC	P-value	Cronbach's alpha	ICC	P-value	Cronbach's alpha	ICC	P-value
Length	1	1	<0.001*	1	1	<0.001*	1	1	<0.001*	0.999	0.999	<0.001*
Depth	1	0.999	<0.001*	1	1	<0.001*	0.999	0.999	<0.001*	1	1	<0.001*
APD	0.999	0.999	<0.001*	1	1	<0.001*	1	1	<0.001*	0.999	0.999	<0.001*

## Results

Statistical analysis

A one-way ANOVA test was used for quantitative data between the three groups followed by a post hoc test between each two groups to compare linear measurements of sella. Also, the independent sample T-test was used for quantitative data between the two groups to compare different measurements between different age groups and sex groups. The significance level was set at P≤0.05. Statistical analysis was performed with IBM SPSS Statistics for Windows, version 23.0 (Armonk, NY, IBM Corp.).

Table 2 showed a significant difference in depth (D) between class I and class II, with class II having a greater depth. Also, the largest diameter (APD) was found in the class III group. No other significant differences in measurements were found between classes.

**Table 2:** Comparison of different measurements between different classes One-way ANOVA test for quantitative data between the three groups followed by post hoc test between each two groups. Superscripts with different small letters refer to significant difference between the two groups. *Significant level at P-value <0.05

		Class I	Class II	Class III	P-value
		N=100	N=100	N=100	
Length (L)	Range mean±SD	(7.4-16.5) 11.8±2.5	(5.9-15.3) 11.8±2	(6.3-15.3) 11.8±1.9	0.962
Depth (D)	Range mean±SD	(3.2-11.5)^a^ 6.6±2	(3.9-13.8)^b^ 7.3±1.9	(3.7-11.4) 7.1±1.9	0.034*
Diameter (APD)	Range mean±SD	(8.4-15.2) 12±1.6	(7.6-16.5) 12.5±1.5	(9.5-16) 12.5±1.6	0.067

Table 3 showed a significant difference in diameter (APD) between the two age groups, with the older age group (25-30 years) having a greater diameter. No significant differences were found for length or depth between age groups.

**Table 3:** Comparison of different measurements between different age groups Independent sample T-test for quantitative data between the two groups. *Significant level at P-value <0.05

		Age		P-value
		18-24 Y	25-30 Y	
		N=169	N=131	
Length (L)	Range mean±SD	(6.3-15.6) 11.7±2	(5.9-16.5) 12±2.3	0.251
Depth (D)	Range mean±SD	(3.7-11.5) 7.1±1.7	(3.2-13.8) 7±2.2	0.638
Diameter (APD)	Range mean±SD	(7.6-15.2) 12.2±1.3	(8.4-16.5) 12.5±1.9	0.039*

In Table 4 (measurements in mm), highly significant differences in length (greater in males) and depth (greater in females) between sexes were found. No significant difference was found in diameter between males and females.

**Table 4:** Comparison of different measurements between different sex Independent sample T-test for quantitative data between the two groups. *Significant level at P-value <0.05

		Sex		P-value
		Male	Female	
		N=108	N=192	
Length (L)	Range mean±SD	(6.3-16.5) 12.4±2.1	(5.9-15.3) 11.4±2.1	<0.001*
Depth (D)	Range mean±SD	(3.7-10.7) 6.5±1.7	(3.2-13.8) 7.3±2	<0.001*
Diameter (APD)	Range mean±SD	(9.3-15.3) 12.4±1.5	(7.6-16.5) 12.3±1.6	0.433

Table 5 showed no significant differences in length, depth, or diameter between the three classes in the 18-24 years age group.

**Table 5:** Comparison of different measurements between different classes in the age group 18-24 years One-way ANOVA test for quantitative data between the three groups followed by post hoc test between each two groups.

In age 18-24		Class I	Class II	Class III	P-value
		N=64	N=51	N=54	
Length (L)	Range mean±SD	(7.4-15.6) 11.6±2.3	(6.3-15.2) 11.5±1.7	(6.3-14.8) 11.9±2	0.622
Depth (D)	Range mean±SD	(4-11.5) 6.8±1.9	(3.9-10) 7.2±1.5	(3.7-10.3) 7.2±1.7	0.372
Diameter (APD)	Range mean±SD	(9.8-13.6) 12±1.2	(7.6-14.3) 12.1±1.3	(9.5-15.2) 12.5±1.3	0.100

Table 6 showed a significant difference in depth between class I and class II in the 25-30 years age group, with class II having greater depth. No other significant differences were found.

**Table 6:** Comparison of different measurements between different classes in the age group 25-30 years One-way ANOVA test for quantitative data between the three groups followed by post hoc test between each two groups. Superscripts with different small letters refer to significant difference between the two groups. *Significant level at P-value <0.05

In age 25-30		Class I	Class II	Class III	P-value
		N=36	N=49	N=46	
Length (L)	Range Mean±SD	(7.5-16.5) 12.3±2.8	(5.9-15.3) 12±2.2	(8.6-15.3) 11.7±1.9	0.494
Depth (D)	Range Mean±SD	(3.2-10.7)^a^ 6.3±2.2	(4.1-13.8)^b^ 7.5±2.2	(4.4-11.4) 7±2.1	0.049*
Diameter (APD)	Range Mean±SD	(8.4-15.2) 12.2±2.1	(10-16.5) 12.8±1.7	(10.2-16) 12.5±1.8	0.290

Table 7 showed no significant differences in length, depth, or diameter between the three classes in males.

**Table 7:** Comparison of different measurements between different classes of males One-way ANOVA test for quantitative data between the three groups followed by post hoc test between each two groups.

In males		Class I	Class II	Class III	P-value
		N=35	N=31	N=42	
Length (L)	Range mean±SD	(7.9-16.5) 12.9±2.6	(9.4-14.4) 11.7±1.3	(6.3-15.3) 12.6±2.1	0.062
Depth (D)	Range mean±SD	(3.7-10.7) 6.4±2	(3.9-9.4) 6.9±1.5	(3.7-8.9) 6.2±1.4	0.164
Diameter (APD)	Range mean±SD	(9.3-15.2) 12.7±1.6	(9.6-14.3) 12.3±1.2	(9.5-15.3) 12.4±1.5	0.494

Table 8 showed a significant difference in depth between classes I and III and in diameter between class I and the other two classes in females. Class III had greater depth and classes II and III had greater diameter than class I.

**Table 8:** Comparison of different measurements between different classes of females One-way ANOVA test for quantitative data between the three groups followed by post hoc test between each two groups. Superscripts with different small letters refer to significant difference between the two groups. *Significant level at P-value <0.05

In females		Class I	Class II	Class III	P-value
		N=65	N=69	N=58	
Length (L)	Range mean±SD	(7.4-14.7) 11.3±2.3	(5.9-15.3) 11.8±2.3	(8.5-14.2) 11.2±1.6	0.254
Depth (D)	Range mean±SD	(3.2-11.5)^a^ 6.8±2	(4-13.8) 7.5±2	(4.4-11.4)^b^ 7.8±1.9	0.009*
Diameter (APD)	Range mean±SD	(8.4-15)^a^ 11.7±1.5	(7.6-16.5)^ b^ 12.6±1.6	(9.5-16)^b^ 12.6±1.7	0.002*

## Discussion

The lateral cephalometric radiographs of 300 patients (108 males and 192 females) from each group (100 patients) with ages ranging from 18 to 30 years old were examined based on the skeletal patterns of classes I, II, and III. The ST was measured in order to determine the linear measurements of its diameter, depth, and length. Each lateral cephalometric radiograph was analyzed. A discrepancy in measurements was observed when comparing the length, depth, and diameter of the ST in this study to those in other studies [1,14,16,17].

The mean linear dimensions of the ST were measured by the researchers and determined to be 8 mm, 6 mm, and 12 mm for the Norwegian population and 9.3 mm, 7.2 mm, and 10.9 mm for the Chinese population for length, depth, and diameter. Also, the average linear dimensions of the Nepalese population were determined to be 8.92 mm, 6.88 mm, and 9.96 mm in length, depth, and diameter. In the current study, the average linear dimensions of ST in upper Egyptian populations were higher than those of the prior research in terms of length, depth, and diameter, 7.3±1.9 mm, 12.5±1.6 mm, and 11.8±2 mm, respectively. On the other hand, these results are consistent with the average transverse dimensions of 10.8 mm, 9.1 mm, and 14 mm for the Saudi populations in terms of length, depth, and diameter.

A noticeable difference was observed when comparing the dimensions of the sella with the skeletal pattern. A notable contrast was observed in the depth of sella when comparing class I and class II individuals. Class II subjects tended to have higher depth values more frequently, while class III subjects generally had the largest diameter values. These results were in agreement with the findings of the previous study and contrasted with the findings of other previous studies, which found no significant relationship between the skeletal pattern and linear measurements of the sella [9,18-20]. Meanwhile, another study found that there was no significant correlation between the average size of the sella and various skeletal types [21]. This might be because they utilized the mean area of sella for the correlation analysis, leading to a different conclusion compared to the linear parameters used in the current study.

The sella diameter of the older age group (12.5±1.9 mm) in the current study was consistently larger than the younger group (12±1.3 mm). Previous studies that discovered a strong relationship between age and sella size also reported similar findings [21,22]. The current investigation supports earlier findings and adds to the body of data indicating that an individual's growth may be evaluated using the size of the ST at various ages. According to the study's findings, forensic medical investigations may find further use for the linear dimensions of ST as hints for determining an individual's age. It was determined whether gender and ST size were correlated. There was a variation seen in sella's linear measures, namely in diameter, depth, and length, among different skeletal patterns. In this study, the males with class III pattern had increased measures in length (12.6±2.1 mm), whereas the females with class II pattern had increased values in depth (7.5±2 mm) and diameter (12.6±1.6 mm) of sella. With the exception of the skeletal class II pattern, the variations in diameter between the male and female groups were not statistically significant. Similar non-significant findings for diameter by gender have been documented in earlier researches [1,3,18]. There was no discernible relationship between gender and ST size in participants from China and Nepal. The study's findings were in line with earlier researches on subjects from India, Saudi Arabia, Pakistan, Malaysia, Bangladesh, Nigeria, Iran, and Iraq [1,23-26]. No discernible difference was found between the different sexual orientations with regard to the ST's linear variables.

By measuring the diameter of the sella, the current study's findings can be very helpful in determining the skeletal pattern of adolescents. Nevertheless, the study had several shortcomings. In order to examine the age-related changes in sella linear dimensions, the younger age group should have been included. A larger sample size would have shown a more substantial connection between the observed parameters.

## Conclusions

According to our findings, the linear measurements of ST in late adolescence and adulthood upper Egyptians show unique differences between gender and skeletal differences, and we can predict different skeletal malocclusion from the dimension of ST. The larger diameter values were seen in the skeletal class III upper Egyptian subjects (12.5±1.6 mm), while the larger depth values were observed in the class II upper Egyptian subjects (7.3±1.9 mm). Highly significant differences were found in length (more in males 12.6±2.1 mm) and depth (more in females 7.5±2 mm) between sexes. The growth of the individuals can be assessed based on the size of the ST at different age periods. The older age group (25-30 years) has a greater diameter in the sella (12.5±1.9 mm) than the younger one (12±1.3 mm) and suggests that the linear dimensions of ST can be used as additional clues for age determination in forensic medical investigations.
